# Pharmacokinetic diversity of baloxavir marboxil across rare avian species

**DOI:** 10.1371/journal.pone.0361119

**Published:** 2026-10-23

**Authors:** Yo Shimazu, Ryo Daniel Obara, Mariko Miki, Hiromi Osaki, Yuto Yoshimoto, Marina Hayashi, Kosuke Toyoda, Miki Ozaki, Ikumi Hanada, Rei Matsumoto, Kaori Ota, Ayumi Yamaguchi, Kosuke Toda, Junko Ishii, Tsuneo Kizaki, Yuzo Hirano, Yuma Sugimoto, Yukiko Watanabe, Keisuke Saito, Masato Kaku, Izumi Ide, Akihiro Sado, Hidefumi Sakai, Kyohei Nishimura, Mayumi Endo, Takahiro Hiono, Norikazu Isoda, Takao Shishido, Yoshinori Ikenaka, Yoshihiro Sakoda

**Affiliations:** 1 Laboratory of Microbiology, Faculty of Veterinary Medicine, Hokkaido University, Sapporo, Hokkaido, Japan; 2 Laboratory for Drug Discovery and Development, Shionogi and Co., Ltd., Toyonaka, Osaka, Japan; 3 Tokyo Sea Life Park, Edogawa-ku, Tokyo, Japan; 4 Adventure World, Nishimuro-gun, Wakayama, Japan; 5 Hyogo Park of the Oriental White Stork, Toyooka, Hyogo, Japan; 6 Tama Zoological Park, Hino, Tokyo, Japan; 7 Tokyo Zoological Park Society, Taito-ku, Tokyo, Japan; 8 Ueno Zoological Gardens, Taito-ku, Tokyo, Japan; 9 Institute for Raptor Biomedicine Japan, Kushiro, Hokkaido, Japan; 10 Shikoku Aquarium, Utazu-cho, Kagawa, Japan; 11 Toyama Outdoor Nature Museum “Nei no Sato”, Toyama, Toyama, Japan; 12 Sapporo Maruyama Zoo, Sapporo, Hokkaido, Japan; 13 One Health Research Center, Hokkaido University, Sapporo, Hokkaido, Japan; 14 International Institute for Zoonosis Control, Hokkaido University, Sapporo, Hokkaido, Japan; 15 Institute for Vaccine Research and Development (HU-IVReD), Hokkaido University, Sapporo, Hokkaido, Japan; 16 Laboratory for Discovery and Disease Research, Shionogi and Co., Ltd., Toyonaka, Osaka, Japan; 17 Laboratory of Toxicology, Department of Environmental Veterinary Science, Faculty of Veterinary Medicine, Hokkaido University, Sapporo, Hokkaido, Japan; 18 Translational Research Unit, Veterinary Teaching Hospital, Faculty of Veterinary Medicine, Hokkaido University, Sapporo, Hokkaido, Japan; 19 Water Research Group, Unit for Environmental Sciences and Management, North-West University, Potchefstroom, South Africa; National Institute of High Security Animal Diseases of Indian Council of Agricultural Research: National Institute of High Security Animal Diseases, INDIA

## Abstract

High pathogenicity avian influenza virus (HPAIV) poses a serious threat to captive rare birds. Because effective countermeasures are limited, the use of human antiviral drugs to treat HPAIV infections in captive rare birds has been considered. Although the human antiviral drug, baloxavir marboxil (BXM), is not approved for the treatment of influenza virus infection in birds, previous studies demonstrated the antiviral efficacy of BXM in experimental avian models. Pharmacokinetic studies have also revealed substantial interspecies differences following BXM administration, suggesting the need to establish species-specific dosing regimens. In this study, BXM was administered to 14 avian species to optimize dosing regimens for each species. Oral administration of BXM using different methods revealed that drug–food interactions had a limited impact on systemic drug exposure. Pharmacokinetic analysis of BXM and its active metabolite, baloxavir acid (BXA), demonstrated marked interspecies variability in drug exposure, contributing to the development of optimized dosing regimens for each species. Furthermore, systemic exposure to BXA was strongly associated with drug absorption indicators, suggesting that species-specific differences in absorption efficiency largely determine BXA exposure among avian species. This study provides a pharmacokinetic basis for the rational use of BXM in diverse avian species. It contributes to the development of practical antiviral strategies to protect captive rare birds against HPAIVs.

## 1. Introduction

High pathogenicity avian influenza viruses (HPAIVs) cause severe and often fatal disease in a wide range of avian species [1]. Since 2020, H5 HPAIVs belonging to HA clade 2.3.4.4b have been widely disseminated throughout wild bird populations, causing extensive outbreaks in poultry and substantial mortality in wild birds [2]. This ongoing panzootic has also affected rare avian species across multiple regions, including Europe, Asia, Africa, the Americas, and Antarctica [3,4]. Despite the ongoing risk of HPAIV infection, birds in zoological institutions are often housed in open enclosures to meet animal welfare requirements, which inherently limits the implementation of strict biosecurity measures. Consequently, zoos have experienced HPAIV outbreaks among captive birds, leading to the loss of rare avian species, such as snowy owls (*Bubo scandiacus*), black swans (*Cygnus atratus*), and Humboldt penguins (*Spheniscus humboldti*) [5,6]. Although some European countries have implemented vaccination programs against HPAIV in captive birds, vaccine efficacy varies among species [7]. In countries where vaccination is not implemented for poultry, such as Japan, controlling HPAIV infection in both zoos and poultry farms relies primarily on enhanced biosecurity measures [8]. The standard recommendation when HPAIV infection is confirmed in captive flocks is the culling of infected individuals and contact birds to prevent further spread and eliminate the virus. However, from the standpoint of conservation medicine, the euthanasia of endangered avian species may not be desirable. Accordingly, therapeutic options for HPAIV infections in captive rare birds have been explored.

Several anti-influenza drugs, originally developed for human influenza, have been evaluated for the treatment of HPAIV infections in a chicken model. Human anti-influenza drugs are classified into neuraminidase (NA) and polymerase acidic protein (PA) inhibitors [9,10]. NA, a viral surface glycoprotein with sialidase activity, plays a role in facilitating the release of progeny virions from infected host cells. Evaluation of NA inhibitors, such as oseltamivir, zanamivir, and peramivir, in HPAIV-infected chickens revealed that none of them completely averted disease onset or mortality [11,12]. This limited efficacy may reflect that NA inhibitors primarily suppress viral release without inhibiting viral replication. In contrast, PA inhibitors act during viral RNA transcription by inhibiting the PA subunit of the influenza virus RNA-dependent RNA polymerase complex, which possesses the endonuclease activity responsible for cap-snatching, a process essentially required for viral mRNA transcription [13]. Baloxavir marboxil (BXM) is a PA inhibitor approved for clinical use in humans and is an orally administered prodrug that is converted to its pharmacologically active form, baloxavir acid (BXA) [14]. BXA treatment inhibited the replication of H5 HPAIVs in vitro, while oral administration of BXM suppressed viral replication and improved survival in an H5N1-infected mouse model [15]. In chickens that exhibit extremely rapid disease progression and typically die within 2–3 days after HPAIV infection, prophylactic administration of BXM at 2.5 mg/kg at the time of HPAIV inoculation was sufficient to prevent mortality [12]. However, in chickens, administration initiated one day after infection failed to confer therapeutic benefits, even at substantially higher doses. This limited efficacy is likely attributable to the narrow therapeutic window imposed by the rapid disease progression in this species [16]. In contrast to chickens, many avian species, including ducks, sparrows, crows, herons, white-tailed eagles, and wild waterfowl, exhibit a prolonged disease course following HPAIV infection, with clinical progression occurring over several days [17–20]. In ducks, which typically die 4–7 days after HPAIV infection, BXM treatment was initiated 48 hours (h) post-infection and continued at 2.5 mg/kg for 5 consecutive days, effectively reducing both mortality and virus shedding [21]. These findings suggest that therapeutic administration of BXM after infection may be feasible in avian species with slower disease progression. In that study, systemic exposure to BXA, as measured by the area under the concentration–time curve (AUC), correlated with these therapeutic effects, suggesting that maintaining BXA exposure above a certain threshold is critical for antiviral efficacy. These findings provide a basis for considering the application of BXM treatment to rare avian species affected by HPAIV.

Despite these findings, the safety and pharmacokinetics of BXM in avian species have not yet been fully characterized. Although no clinical signs or organ toxicity were observed in chickens, even at doses up to 62.5 mg/kg administered for 28 consecutive days, corresponding to a 71-fold safety margin, the species-specific drug toxicity of BXM cannot be excluded, as exemplified by the severe toxicity of diclofenac, a nonsteroidal anti-inflammatory drug, in vulture species [22,23]. Moreover, the pharmacokinetic profiles of drugs vary considerably among the different avian species. For example, the elimination half-life of carboplatin, a platinum-based antineoplastic agent, is correlated with body weight across avian species, whereas that of amikacin, an aminoglycoside antibiotic, shows little species variation [24,25]. In contrast, the pharmacokinetic properties of opioids are significantly influenced by the species [26]. Therefore, a direct evaluation of the pharmacokinetic profile of each target species is essential to establish appropriate dosing regimens. In addition, BXM absorption is reduced by food intake in humans, raising the possibility that species-specific feeding habits and administration conditions influence BXA exposure in birds [27]. Furthermore, as clinical use in birds would likely require repeated administration over several days, it is necessary to evaluate the potential safety and drug accumulation under repeated-dose regimens.

In the present study, the effects of different oral administration methods on the pharmacokinetics of BXA were evaluated in white-tailed eagles (*Haliaeetus albicilla*) to assess the influence of feeding conditions on systemic BXA exposure. Subsequently, a comprehensive pharmacokinetic evaluation following BXM administration was conducted in a diverse range of rare avian species in Japan. In addition, a repeated-dose study was conducted in a Eurasian eagle-owl (*Bubo bubo*) to evaluate the potential drug accumulation associated with consecutive dosing. Based on the pharmacokinetic data obtained for these species, practical and species-optimized dosing regimens using commercially available BXM formulations were proposed for therapeutic use in infected individuals and potential metaphylactic use in exposed birds following detection of HPAIV infection within a flock or facility.

## 2. Materials and methods

### 2.1. Animals and drugs

#### 2.1.1. Animals.

Fourteen avian species housed in zoos, aquariums, and wildlife rehabilitation centers in Japan were used in this study. The study species were selected based on their conservation status or on reported cases of HPAIV infection. Among the study species, HPAIV infections have been reported in wild birds in Japan, including several species of conservation concern according to the Red List of the Ministry of the Environment, Japan: common murres (*Uria aalge*; Critically Endangered [CR]), Oriental storks (*Ciconia boyciana*; Endangered [EN]), black-faced spoonbills (*Platalea minor*; Vulnerable [VU]), white-tailed eagles (Vulnerable [VU]), and peregrine falcons (*Falco peregrinus*; Near Threatened [NT]) [28]. Puna ibis (*Plegadis ridgwayi*) was included as a species closely related to the crested ibis (*Nipponia nippon*), which is classified as Endangered (EN), to provide comparative pharmacokinetic information within the same taxonomic group. The rock ptarmigan included in this study was the Svalbard rock ptarmigan (*Lagopus muta hyperborea* Sundevall), a subspecies of the same species as the Japanese rock ptarmigan (*Lagopus muta japonica* Clark), which is classified as Endangered (EN). The Eurasian eagle-owl was included as a species closely related to Blakiston’s fish owl (*Bubo blakistoni*), an Endangered (EN) species in which HPAIV infection has been reported, providing comparative pharmacokinetic information for a taxonomically related species of conservation concern. The endemic Amami jay (*Garrulus lidthi*) was included as a species of conservation importance in Japan, although it is not currently classified as threatened. To reflect the conditions commonly encountered in zoological facilities, several penguin species, including Humboldt penguins (Vulnerable), African penguins (*Spheniscus demersus*; Critically Endangered), and southern rockhopper penguins (*Eudyptes chrysocome*; Vulnerable), according to the IUCN Red List, were included. Humboldt penguins have experienced HPAIV infections in both wild and captive settings, African penguins have been infected with HPAIV in the wild, and southern rockhopper penguins are frequently housed in zoos despite the absence of confirmed HPAIV infections [6,29,30]. Furthermore, the great white pelicans (*Pelecanus onocrotalus*) and American flamingos (*Phoenicopterus ruber*), both of which have experienced HPAIV outbreaks in zoological collections, were included in this study. The number of animals, age, body weight, and sex of each species are summarized in Table 1 and S1 Table. All individuals were confirmed healthy based on clinical observations. Preliminary hematological examinations were also conducted for certain species when samples were available (S2 Table). Throughout the study period, all birds were monitored at least twice daily for clinical signs, including changes in activity, posture, feather condition, respiratory status, and fecal consistency, and abnormal findings were recorded. Particular attention has been paid to signs of diarrhea and anaphylaxis, which have been reported in human clinical trials.

**Table 1:** Species, number of animals, body weight ranges, doses, and administration routes of baloxavir marboxil in the birds included in this study.

Species	Scientific name	Number of animals	Body weight range (g)	Dose (mg/kg)	Route of administration
White-tailed eagle	*Haliaeetus albicilla*	4	4,640–5,360	2.5	Three different routes^*^
Humboldt penguin	*Spheniscus humboldti*	4	4,400–5,020	2.5, 12.5	Encapsulated BXM placed in fish
African penguin	*Spheniscus demersus*	4	2,660–3,430	2.5, 12.5	Powdered BXM placed in fish
Southern rockhopper penguin	*Eudyptes chrysocome*	6	1,720–2,340	2.5, 12.5	Encapsulated BXM placed in fish
Puna ibis	*Plegadis ridgwayi*	3	636–688	2.5, 12.5	Powdered BXM placed in fish
Common murre	*Uria aalge*	5	687–781	2.5, 12.5	Encapsulated BXM placed in fish
Great white pelican	*Pelecanus onocrotalus*	4	5,760–10,350	2.5, 12.5	Encapsulated BXM placed in fish
American flamingo	*Phoenicopterus ruber*	4	2,060–3,200	2.5, 12.5	Suspended BXM in saline via gastric tube
Peregrine falcon	*Falco peregrinus*	2	589–948	2.5, 12.5	Encapsulated BXM placed in meat
Oriental stork	*Ciconia boyciana*	6	3,900–5,500	2.5, 12.5	Encapsulated BXM placed in fish
Black-faced spoonbill	*Platalea minor*	3	1,140–1,548	2.5	Powdered BXM placed in fish
Rock ptarmigan	*Lagopus muta*	3	646–747	12.5	Powdered BXM sprinkled on berries
Amami jay	*Garrulus lidthi*	5	152–186	12.5	Powdered BXM mixed in pellets
Eurasian eagle-owl	*Bubo bubo*	1	3,000	2.5, 5.0, 10	Powdered BXM placed in meat

* First administration, forced feeding of trout containing encapsulated BXM; second administration, forced administration of an oral capsule followed by trout feeding; third administration, gastric tube administration of a BXM suspension.

#### 2.1.2. Drugs.

BXM (20 mg tablets, Shionogi & Co., Ltd., Osaka, Japan) was crushed and weighed based on the body weight of each individual.

### 2.2. Administration experiment

#### 2.2.1. Evaluation of oral administration routes for baloxavir marboxil in white-tailed eagles.

Four white-tailed eagles were administered BXM at 2.5 mg/kg. BXM was administered three times at intervals of at least one week, using a different administration method for each trial. For the first administration, crushed BXM was encapsulated and inserted into the oral cavity of rainbow trout, which were then force-fed to the birds. For the second administration, the encapsulated BXM was manually inserted into the oral cavity, followed immediately by forced feeding of the rainbow trout. For the third administration, the crushed BXM was suspended in water and administered via a gastric tube. After each administration, all birds were monitored for clinical abnormalities potentially attributable to the BXM administration. In all trials, 230 μL of blood was collected at 2, 8, 24, and 48 h post-administration (hpa). The collected blood samples were thoroughly mixed with 13.8 μL of an enzyme inhibitor–anticoagulant mixture (60 mmol/L dichlorvos [FUJIFILM Wako Pure Chemical Corp., Osaka, Japan] and sodium heparin [Nipro Corp., Osaka, Japan]) and centrifuged at 1900 × *g* for 15 min to obtain plasma. Plasma samples were stored at –80°C until further analysis.

#### 2.2.2. Single oral administration of baloxavir marboxil in rare avian species.

BXM was orally administered to Humboldt penguins, African penguins, southern rockhopper penguins, puna ibises, common murres, great white pelicans, American flamingos, peregrine falcons, Oriental storks, and black-faced spoonbills. The routes of administration for each species are summarized in Table 1. These routes were chosen for their practical applicability in animal facilities to ensure safe and effective use even in HPAIV-infected individuals. An initial dose of 2.5 mg/kg was administered to all animals. A second dose of 12.5 mg/kg was administered after an interval of at least two weeks to all species, except black-faced spoonbills. For all species except great white pelicans, 230 μL of blood was collected at 2, 8, 24, and 48 hpa, followed by plasma separation and storage as described above. In great white pelicans, blood sampling was performed at 6, 8, 24, and 48 hpa to minimize the risk of regurgitation during restraint. Additionally, to reduce the physiological burden associated with blood sampling, rock ptarmigans and Amami jays received a single oral dose of BXM at 12.5 mg/kg, with blood samples collected at 2, 8, and 24 hpa under thiopental anesthesia. After each administration, all birds were monitored for clinical abnormalities potentially attributable to the BXM administration.

#### 2.2.3. Repeated administration of baloxavir marboxil in Eurasian eagle-owl.

Powdered BXM at 2.5 mg/kg was mixed with horse meat and orally administered to a Eurasian eagle-owl once daily for 7 consecutive days. Blood samples were collected 2, 8, and 24 h after the initial dose, immediately before the seventh dose, and 2, 8, and 24 h after the seventh dose. After an interval of at least one week between dosing periods, a 5.0 mg/kg regimen was administered, followed by a 10 mg/kg regimen. In the 5.0 mg/kg and 10 mg/kg regimens, blood samples were collected immediately before the initial and seventh doses, as well as at 2, 8, and 24 h after each dose. Plasma separation and storage were performed as previously described. After each administration, the bird was monitored for clinical abnormalities potentially attributable to the BXM administration.

### 2.3. LC–MS/MS analysis of baloxavir marboxil and baloxavir acid

Plasma concentrations of BXM and BXA were determined by liquid chromatography–tandem mass spectrometry (LC–MS/MS) using an Agilent 6495B system (Agilent Technologies, Inc., Santa Clara, CA, USA). For BXM analysis, 10 μL of plasma was mixed with 100 μL of methanol and 100 μL of acetonitrile containing the internal standard. For BXA analysis, 3 μL of plasma was mixed with 200 μL of methanol and 200 μL of internal standard solution. The mixture was vortexed and incubated at –80°C for 30 min, followed by centrifugation at –10°C and 10,000 × *g* for 10 min. The resulting supernatant was mixed with an equal volume of 0.1% EDTA-2K solution (Dojindo Laboratories, Kumamoto, Japan) and analyzed using LC–MS/MS. Stable isotope-labeled internal standards were used for quantification. BXM-d_3_^18^O and BXA-racemate-d_4_^18^O were used as the internal standards for BXM and BXA analyses, respectively. Chromatographic separation was performed using a 2.1 mm × 100 mm L-column3 C18 metal-free column with a particle size of 2.0 μm (Chemicals Evaluation and Research Institute, Tokyo, Japan) maintained at 45°C. Mobile phase A consisted of distilled water containing 0.1% formic acid, and mobile phase B consisted of acetonitrile containing 0.1% formic acid. The analytes were separated at a flow rate of 0.3 mL/min. The gradient program was as follows: the initial condition was 45% B, which was increased linearly to 70% B from 0.2 min to 4 min, followed by 100% B for 1 min. The system was returned to 45% B and equilibrated for 1 min before the next injection. The injection volumes were 10 μL for BXM and 3 μL for BXA. Detection was performed using electrospray ionization in the positive mode. The retention time (RT), multiple reaction monitoring (MRM) transition, and collision energy (CE) for each analyte were as follows: BXM, 2.72 min, 572.2 → 214.0, 80.0 eV; BXM-d_3_^18^O, 3.40 min, 577.1 → 214.0, 68.0 eV; BXA, 2.23 min, 484.1 → 246.9, 20.0 eV; and BXA-racemate-d_4_^18^O, 2.20 min, 490.1 → 247.2, 24.0 eV. BXM and BXA were quantified using the internal standard method based on the ratio of the analyte peak area to that of the internal standard. The lower limit of quantification (LLOQ) was 4 ng/mL for both BXM and BXA. Calibration standards and quality control samples were prepared using blank plasma obtained from each bird species to minimize potential matrix effects. A calibration curve consisting of at least six concentration points over the range 4–2000 ng/mL was prepared and used for quantification. The calibration curves showed coefficients of determination (*R*^2^) > 0.99. The QC samples were prepared at low, medium, and high concentrations within the calibration range, and their accuracy was confirmed to be 85–115%.

### 2.4. Pharmacokinetic analysis

Pharmacokinetic parameters, including the maximum plasma concentration (C_max_), area under the plasma concentration–time curve from 0–12 h (AUC_0–12h_), 0–24 h (AUC_0–24h_), 24–48 h (AUC_24–48h_), and elimination rate constant (k_el_), were determined using the linear trapezoidal method in PKanalix 2024R1 (Simulations Plus, Lancaster, CA, USA). Plasma half-life (T_1/2_) was calculated as ln(2)/k_el_.

### 2.5. Correlation analysis of pharmacokinetic parameters and intestinal morphology

To investigate the factors influencing BXA exposure, the AUC_0–24h_ obtained after oral administration at 2.5 mg/kg was used as the dependent variable (y-axis), and body weight, C_max_, and T_1/2_ were evaluated as independent variables (x-axis). The correlation coefficients were calculated for each pair of variables. In addition, to explore whether interspecies differences in gastrointestinal morphology contribute to the variability in oral drug absorption, correlations between body-weight-adjusted intestinal length indices and pharmacokinetic parameters of BXA following oral administration of BXM at 2.5 mg/kg were analyzed. In birds, the total intestinal length has been reported to scale with body weight to a power of 0.43 [31]. Based on this report, previously reported values for total intestinal length were divided by body weight raised to a power of 0.43 to calculate body weight-adjusted intestinal indices. In addition to the data obtained in the present study, previously reported pharmacokinetic parameters for ducks (*Anas platyrhynchos* var. *domesticus*) and the Okinawa rail (*Hypotaenidia okinawae*) following oral administration at 2.5 mg/kg were included in the analysis [21,32]. When anatomical data were unavailable for the species used in this study, data from species within the same genus or tribe were used as substitutes. Specifically, data for the following species were substituted with those from their respective congeners: white-tailed eagle with red kite (*Milvus milvus*), puna ibis with glossy ibis (*Plegadis falcinellus*), great white pelican with brown pelican (*Pelecanus occidentalis*), Oriental stork with Storm’s stork (*Ciconia stormi*), black-faced spoonbill with roseate spoonbill (*Platalea ajaja*), and duck with mallard (*Anas platyrhynchos*). For Okinawa rail, data from the clapper rail (*Rallus longirostris*) were used, as these species were historically classified within the same genus. However, such anatomical data were unavailable for Humboldt and African penguins; therefore, these species were excluded from the correlation analysis. The mean body-weight-adjusted intestinal indices were treated as independent variables (x-axis), and the dependent variable (y-axis) was the AUC_0–24h_ of BXA. The relationship between AUC_0–24h_ and each pharmacokinetic parameter was analyzed using linear regression models.

### 2.6. Statistical analysis

The mean, standard deviation, median, and quartiles of the plasma concentrations and pharmacokinetic parameters were calculated using Microsoft Excel (Microsoft, Redmond, WA, USA). A two-tailed Student’s t-test was performed to compare pharmacokinetic parameters between dosing routes and to compare parameters obtained for each bird species with those obtained in a previous duck experiment. Linear regression analyses were performed using Microsoft Excel to examine the relationship between individual AUC_0–24h_ and body weight or other pharmacokinetic parameters. When data were unavailable, the corresponding data points were excluded from the analyses. The statistical significance of the correlations was assessed using a test for zero correlation. Differences or correlations with *p* < 0.05 were considered statistically significant.

### 2.7. Ethics statement

All animal experiments were reviewed and approved by the Institutional Animal Care and Use Committee of the Faculty of Veterinary Medicine, Hokkaido University (approval no. 22-0121, approved on 2 December 2022 [Eurasian eagle-owl]; approval no. 24-0062, approved on 17 April 2024 [Humboldt penguin, African penguin, southern rockhopper penguin, puna ibis, great white pelican, American flamingo, peregrine falcon, black-faced spoonbill, rock ptarmigan, and Amami jay]; approval no. 24-0081, approved on 12 June 2024 [white-tailed eagle]; approval no. 24-0110, approved on 4 September 2024 [Oriental stork]). Experiments involving species designated as nationally rare wild fauna and flora were conducted after obtaining approval from the relevant governmental authorities. All experiments were conducted in accordance with institutional guidelines. Since 2007, the Faculty of Veterinary Medicine of Hokkaido University has been accredited by the Association for the Assessment and Accreditation of Laboratory Animal Care International. All personnel involved in the animal experiments completed the institutionally required education and training programs for animal experimentation and welfare. No animals were euthanized as part of this study. Anesthesia was used only where specifically described in the experimental procedures. No analgesics or other pain-relieving treatments were administered unless otherwise stated, because the procedures were considered to cause only minimal and transient discomfort and were not expected to cause pain requiring analgesia. Efforts to minimize animal burden included limiting blood sampling where necessary and monitoring all birds for clinical abnormalities throughout the study. All procedures described above were included in the animal experimental protocols reviewed and approved by the Institutional Animal Care and Use Committee of the Faculty of Veterinary Medicine, Hokkaido University.

## Results

### Effect of administration routes on BXA exposure in white-tailed eagles

BXM was administered to four white-tailed eagles via three different oral routes, and the post-administration health status and route-dependent differences in BXA exposure were evaluated. BXA was used as the primary analyte because it is the pharmacologically active form of BXM and its systemic exposure is associated with antiviral efficacy. No abnormalities in clinical signs attributable to BXM administration were observed for at least one month after dosing via any of the three routes. Comparison of systemic BXA exposure revealed that the AUC_0–24h_ after the first administration (forced feeding of trout containing encapsulated BXM) was significantly higher than that after the second (forced administration of an oral capsule followed by trout feeding) and third (gastric tube administration of a BXM suspension) administrations (*p* = 0.034 and 0.026, respectively). When WTE #3, which showed exceptionally high BXA exposure after the first administration, was excluded from the analysis, the differences in AUC_0–24h_ between the first and second administrations and between the first and third administrations were no longer statistically significant (*p* = 0.14 and 0.11, respectively). No significant differences were observed in the C_max_ or C_24h_ among the three administration routes (*p* > 0.05) (Fig 1).

**Fig 1:**
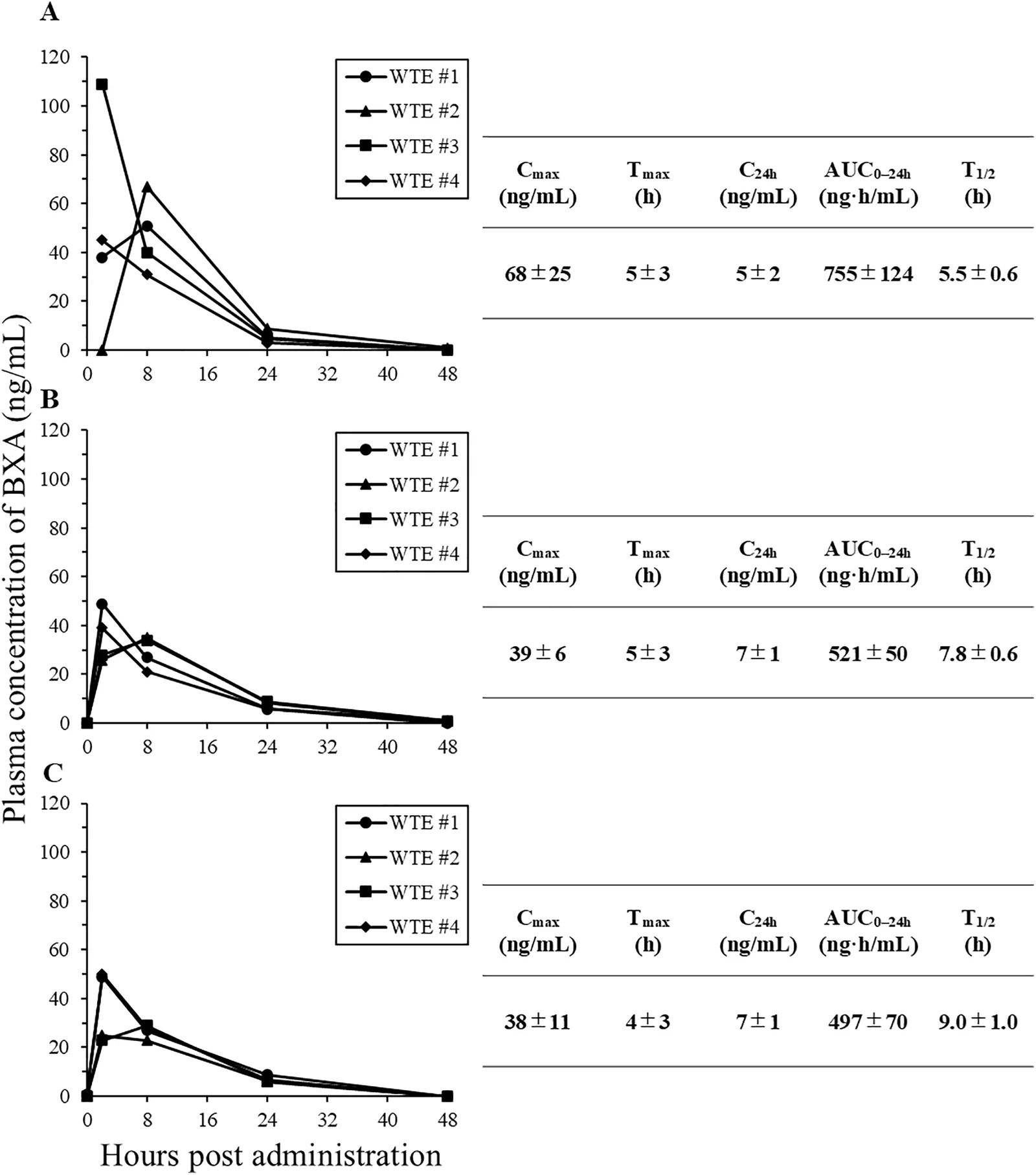
Plasma concentration–time profiles of baloxavir acid (BXA) in white-tailed eagles following oral administration of baloxavir marboxil at 2.5 mg/kg via three different administration routes. (A) Powdered BXM encapsulated in a gelatin capsule, placed in fish and administered by force-feeding. (B) Encapsulated powdered BXM administered by force-feeding followed by feeding with fish. (C) BXM suspended in water and administered via gastric tube. Mean ± standard deviation pharmacokinetic parameters are shown for each administration route.

### Pharmacokinetics of baloxavir marboxil and baloxavir acid after single oral administration of BXM in captive rare birds

BXM was orally administered as a single dose of 2.5 or 12.5 mg/kg to Humboldt penguins, African penguins, southern rockhopper penguins, puna ibises, common murres, great white pelicans, American flamingos, peregrine falcons, Oriental storks, black-faced spoonbills, rock ptarmigans, and Amami jays. No abnormalities in general health status attributable to BXM administration were observed in any of the species. Following the administration of 2.5 mg/kg, marked interspecies differences were observed in the pharmacokinetics of BXA (Fig 2, Table 2). AUC_0–24h_ exceeded 2,374 ng·h/mL in Humboldt penguins, African penguins, southern rockhopper penguins, common murres, American flamingos, and black-faced spoonbills, which was associated with complete prevention of mortality and rapid suppression of virus shedding [21]. In southern rockhopper penguins, the AUC_24–48h_ value exceeded the target level. In the great white pelican administered BXM at 2.5 mg/kg, the AUC_0–24h_ value of 2,038 ng·h/mL was comparable to the target value of 2,374 ng·h/mL observed in ducks (*p* = 0.441). In contrast, the puna ibis, peregrine falcon, and Oriental stork showed lower AUC_0–24h_ values than the target value observed in ducks (*p* = 0.082, 0.007, and 0.008, respectively). C_max_ values were relatively high in Humboldt penguins, southern rockhopper penguins, common murres, American flamingos, and black-faced spoonbills, exceeding 300 ng/mL. In contrast, notably low C_max_ values, below 100 ng/mL, were observed in peregrine falcons and Oriental storks. The median T_1/2_ across all bird species was 8.8 h (IQR, 7.2–11.0 h). Puna ibises and American flamingos showed a relatively short T_1/2_ of 6.1 h, whereas that of southern rockhopper penguins was longer (29.6 h). When BXM was administered at 12.5 mg/kg, both the C_max_ and AUC_0–24h_ of BXA increased in a dose-dependent manner in all animals (Fig 3, Table 3). The AUC_0–24h_ in puna ibises and Oriental storks exceeded or was comparable to the target value of 2,374 ng·h/mL observed in ducks (*p* = 0.474 and 0.750, respectively). In rock ptarmigans, AUC_0–24h_ was still lower than the target value of 2,374 ng·h/mL (*p* = 0.175). However, twice the AUC_0–12h_ (1,255 ng·h/mL × 2 = 2,510 ng·h/mL) exceeded the target value of 2,374 ng·h/mL. Even the 12.5 mg/kg administration did not result in sufficient exposure to BXA in peregrine falcons and Amami jays. These results revealed remarkable interspecies differences in BXA exposure following oral BXM administration, which are relevant for subsequent dosing strategies. Although BXA exposure was the primary focus for developing potential dosing regimens, plasma BXM concentrations were also evaluated to characterize interspecies differences in the pharmacokinetics of the prodrug. The pharmacokinetic parameters of BXM obtained across the three study designs are summarized in Table 4. In all bird species except Humboldt and African penguins, the plasma concentrations of BXM were significantly lower than those of BXA. In contrast, for these two penguin species, the C_max_ of BXM exceeded that of BXA. Overall, BXM tended to be present at low plasma concentrations relative to BXA in most avian species; however, clear interspecies differences were evident following BXM exposure.

**Table 2:** Pharmacokinetic parameters of baloxavir acid in birds administered baloxavir marboxil at 2.5 mg/kg.

Animal species	C_max_ (ng/mL)	T_max_ (h)	C_24h_ (ng/mL)	AUC_0–24h_ (ng·h/mL)	AUC_0–12h_ (ng·h/mL)	AUC_24–48h_ (ng·h/mL)	T_1/2_ (h)
Humboldt penguin	300 ± 13	2	65 ± 25	3,370 ± 479	2,206 ± 166	885 ± 357	10.3 ± 1.1
African penguin	269 ± 47	2	72 ± 15	4,101 ± 773	2,559 ± 469	1,000 ± 196	9.2 ± 0.6
Southern rockhopper penguin	763 ± 136	2	321 ± 86	10,767 ± 2,663	6,255 ± 1,351	5,867 ± 1,706	29.6 ± 12.9
Puna ibis	188 ± 102	2	5 ± 2	1,140 ± 594	962 ± 515	62 ± 29	6.1 ± 3.2
Common murre	671 ± 454	2	58 ± 51	6,061 ± 4,109	4,434 ± 2,957	782 ± 639	7.6 ± 0.6
Great white pelican	147 ± 31	7 ± 1	40 ± 6	2,038 ± 329	1,149 ± 218	614 ± 88	11.3 ± 0.4
American flamingo	328 ± 99	2	26 ± 5	3,315 ± 414	2,425 ± 325	312 ± 55	6.1 ± 0.4
Peregrine falcon	9 ± 1	2	0	77 ± 3	60 ± 2	0	ND
Oriental stork	16 ± 11	7 ± 11	4 ± 2	211 ± 121	135 ± 84	57 ± 33	12.2 ± 2.2
Black-faced spoonbill	354 ± 198	2	354 ± 198	3,851 ± 2,336	2,686 ± 1,579	547 ± 398	7.6 ± 0.2

C_max_, maximum plasma concentration; T_max_, time to reach C_max_; C_24h_, plasma concentration at 24 hours after administration; AUC_0–24h_, area under the plasma concentration–time curve from 0 to 24 hours; AUC_0–12h_, area under the plasma concentration–time curve from 0 to 12 hours; AUC_24–48h_, area under the plasma concentration–time curve from 24 to 48 hours; T_1/2_, terminal elimination half-life; ND, not determined. Data are expressed as mean ± standard deviation.

**Table 3:** Mean ± standard deviation pharmacokinetic parameters of baloxavir acid in birds administered baloxavir marboxil at 12.5 mg/kg.

Animal species	C_max_ (ng/mL)	T_max_ (h)	C_24h_ (ng/mL)	AUC_0–24h_ (ng·h/mL)	AUC_0–12h_ (ng·h/mL)	AUC_24–48h_ (ng·h/mL)	T_1/2_ (h)
Humboldt penguin	995 ± 163	4 ± 3	243 ± 98	13,593 ± 4,520	8,213 ± 2,511	3,462 ± 1,352	9.6 ± 0.7
African penguin	825 ± 211	6 ± 3	181 ± 26	11,199 ± 2,111	6,654 ± 1,671	2,665 ± 211	9.9 ± 1.7
Southern rockhopper penguin	3,845 ± 1,876	3 ± 2	1,486 ± 857	54,382 ± 26,967	32,143 ± 15,137	27,735 ± 17,731	23.6 ± 7.3
Puna ibis	365 ± 61	2	41 ± 46	3,100 ± 1,471	2,257 ± 766	528 ± 609	5.8 ± 1.9
Common murre	4,984 ± 2,970	2	1,050 ± 791	64,136 ± 41,102	41,130 ± 25,632	14,252 ± 10,513	9.5 ± 1.9
Great white pelican	1,255 ± 173	8 ± 1	561 ± 81	20,546 ± 2,619	10,755 ± 1,518	8,682 ± 1,658	13.9 ± 2.9
American flamingo	844 ± 575	2	55 ± 59	6,581 ± 5,205	5,038 ± 3,768	699 ± 775	5.5 ± 1.0
Peregrine falcon	91 ± 22	2	6 ± 4	820 ± 60	608 ± 86	72 ± 42	5.9 ± 1.9
Oriental stork	191 ± 76	2	32 ± 14	2,211 ± 774	1,485 ± 515	488 ± 216	11.0 ± 1.7
Rock ptarmigan	252 ± 98	2	6 ± 4	1,471 ± 610	1,255 ± 506	37 ± 12	4.3 ± 1.2
Amami jay	2 ± 1	6 ± 9	1 ± 1	20 ± 9	12 ± 7	ND	ND

C_max_, maximum plasma concentration; T_max_, time to reach C_max_; C_24h_, plasma concentration at 24 hours after administration; AUC_0–24h_, area under the plasma concentration–time curve from 0 to 24 hours; AUC_0–12h_, area under the plasma concentration–time curve from 0 to 12 hours; AUC_24–48h_, area under the plasma concentration–time curve from 24 to 48 hours; T_1/2_, terminal elimination half-life; ND, not determined. Data are expressed as mean ± standard deviation.

**Table 4:** Pharmacokinetic parameters of baloxavir marboxil in birds administered at 2.5 or 12.5 mg/kg.

Animal species	Dose (mg/kg)	C_max_ (ng/mL)	T_max_ (h)	C_24h_ (ng/mL)	AUC_0–24h_ (ng·h/mL)
White-tailed eagle	2.5 (1st)	3 ± 2	2	0	11 ± 9
	2.5 (2nd)	13 ± 9	17 ± 18	0	126 ± 122
	2.5 (3rd)	28 ± 15	4 ± 3	1 ± 1	271 ± 206
Humboldt penguin	2.5	718 ± 323	2	3.3 ± 2.3	3,032 ± 1,305
	12.5	2,211 ± 1,566	2	2 ± 2	14,298 ± 8,152
African penguin	2.5	321 ± 20	2	0	1,306 ± 87
	12.5	1,214 ± 577	2	0	6,923 ± 2,022
Southern rockhopper penguin	2.5	176 ± 153	3 ± 2	1 ± 1	884 ± 636
	12.5	193 ± 241	2	2 ± 2	864 ± 972
Puna ibis	2.5	0	ND	0	0
	12.5	0	ND	0	0
Common murre	2.5	21 ± 25	2 ± 3	0	150 ± 204
	12.5	80 ± 107	14 ± 17	0	810 ± 1,222
Great white pelican	2.5	3 ± 4	6	0	12 ± 14
	12.5	1 ± 1	6	0	8 ± 8
American flamingo	2.5	41 ± 30	2	0	182 ± 131
	12.5	160 ± 228	2	0	664 ± 938
Peregrine falcon	2.5	18 ± 18	2	0	144 ± 72
	12.5	1 ± 1	2	0	3 ± 3
Oriental stork	2.5	5 ± 5	2	0	25 ± 24
	12.5	104 ± 63	2	1 ± 1	478 ± 272
Black-faced spoonbill	2.5^*^	6 ± 6	2	0	25 ± 24
Rock ptarmigan	12.5^*^	0	ND	0	0
Amami jay	12.5^*^	0	ND	0	0
Eurasian eagle-owl	2.5	0	ND	0	0
	5.0	0	ND	0	0
	10	0	ND	0	0

C_max_, maximum plasma concentration; T_max_, time to reach C_max_; C_24h_, plasma concentration at 24 hours after administration; AUC_0–24h_, area under the plasma concentration–time curve from 0 to 24 hours; *, Either 2.5 or 12.5 mg/kg was administered to minimize the burden on the animal. Data are expressed as mean ± standard deviation.

**Fig 2:**
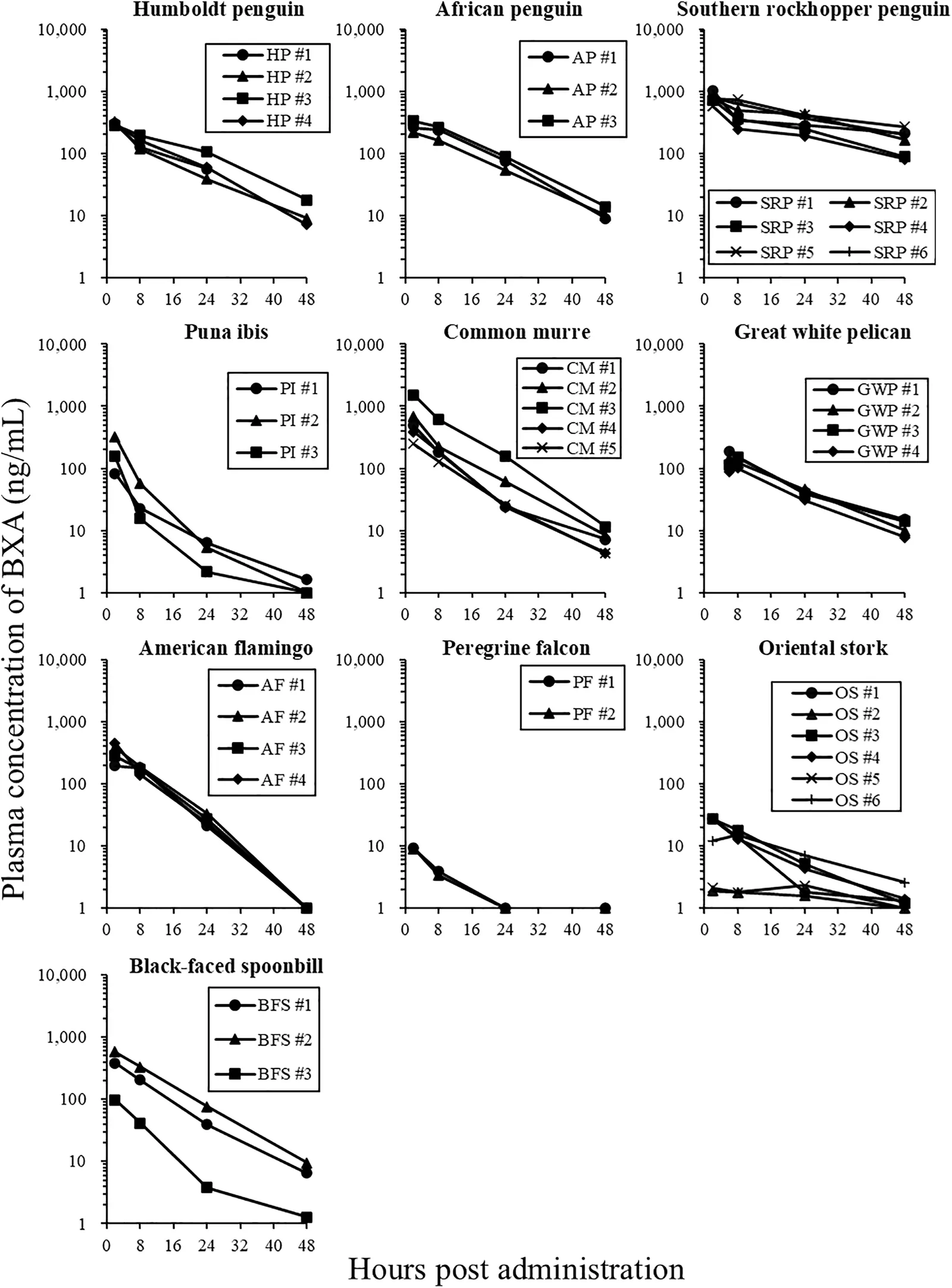
Plasma concentration–time profiles of baloxavir acid (BXA) in captive birds following administration of baloxavir marboxil at 2.5 mg/kg. Each panel represents a different avian species, and each line represents an individual animal.

**Fig 3:**
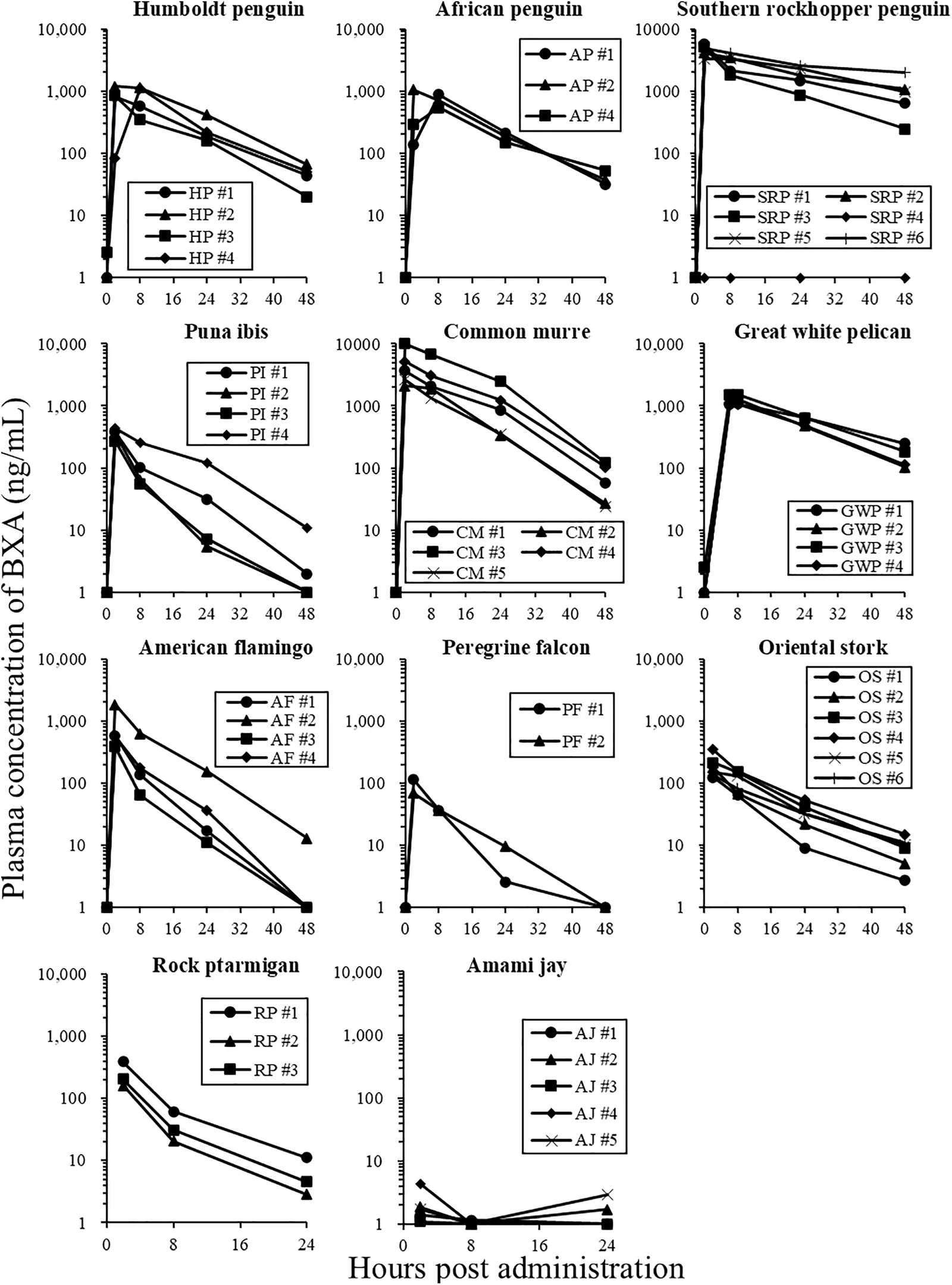
Plasma concentration–time profiles of baloxavir acid (BXA) in captive birds following administration of baloxavir marboxil (BXM) at 12.5 mg/kg. Each panel represents a different avian species, and each line represents an individual animal.

### Repeated administration of BXM in Eurasian eagle-owl

BXM was orally administered to a Eurasian eagle-owl once daily for 7 consecutive days at doses of 2.5, 5.0, and 10 mg/kg to analyze the accumulation of BXA due to consecutive administration. No abnormalities in the general health status attributable to BXM administration were observed throughout the experiment. After the first administration, AUC_0–24h_ values for BXA were 8,371 ng·h/mL (2.5 mg/kg) and 19,181 ng·h/mL (10 mg/kg), and C_max_ values of BXA were 867 ng/mL (2.5 mg/kg), 958 ng/mL (5.0 mg/kg), and 1,190 ng/mL (10 mg/kg), demonstrating a dose-dependent increase (S1 Fig). Following the seventh administration, AUC_0–24h_ values were 11,041 ng·h/mL (2.5 mg/kg) and 20,441 ng·h/mL (10 mg/kg), and C_max_ values were 831 ng/mL (2.5 mg/kg), 1,305 ng/mL (5.0 mg/kg), and 1,574 ng/mL (10 mg/kg). In the Eurasian eagle-owl, although this observation was based on a single individual, a comparison of plasma concentrations after the first and seventh administrations indicated no marked accumulation of BXA during repeated dosing.

### Analysis of anatomical parameters influencing pharmacokinetics of baloxavir acid

To identify the factors influencing BXA exposure, linear regression analyses were performed to examine the relationships between the AUC_0–24h_ of BXA and body weight, C_max_, T_1/2_, and body weight-adjusted total intestinal length in birds administered BXM at 2.5 mg/kg (Fig 4). Body weight showed only a weak correlation with the AUC_0–24h_ (*R*^2^ = 0.149, *p* = 0.006). In contrast, C_max_ showed a strong positive correlation with AUC_0–24h_ (*R*^2^ = 0.864, *p* < 0.001). T_1/2_ and body weight-adjusted total intestinal lengths showed weak correlations with AUC_0–24h_ (*R*^2^ = 0.328, *p* < 0.001 and *R*^2^ = 0.443, *p* < 0.001, respectively).

**Fig 4:**
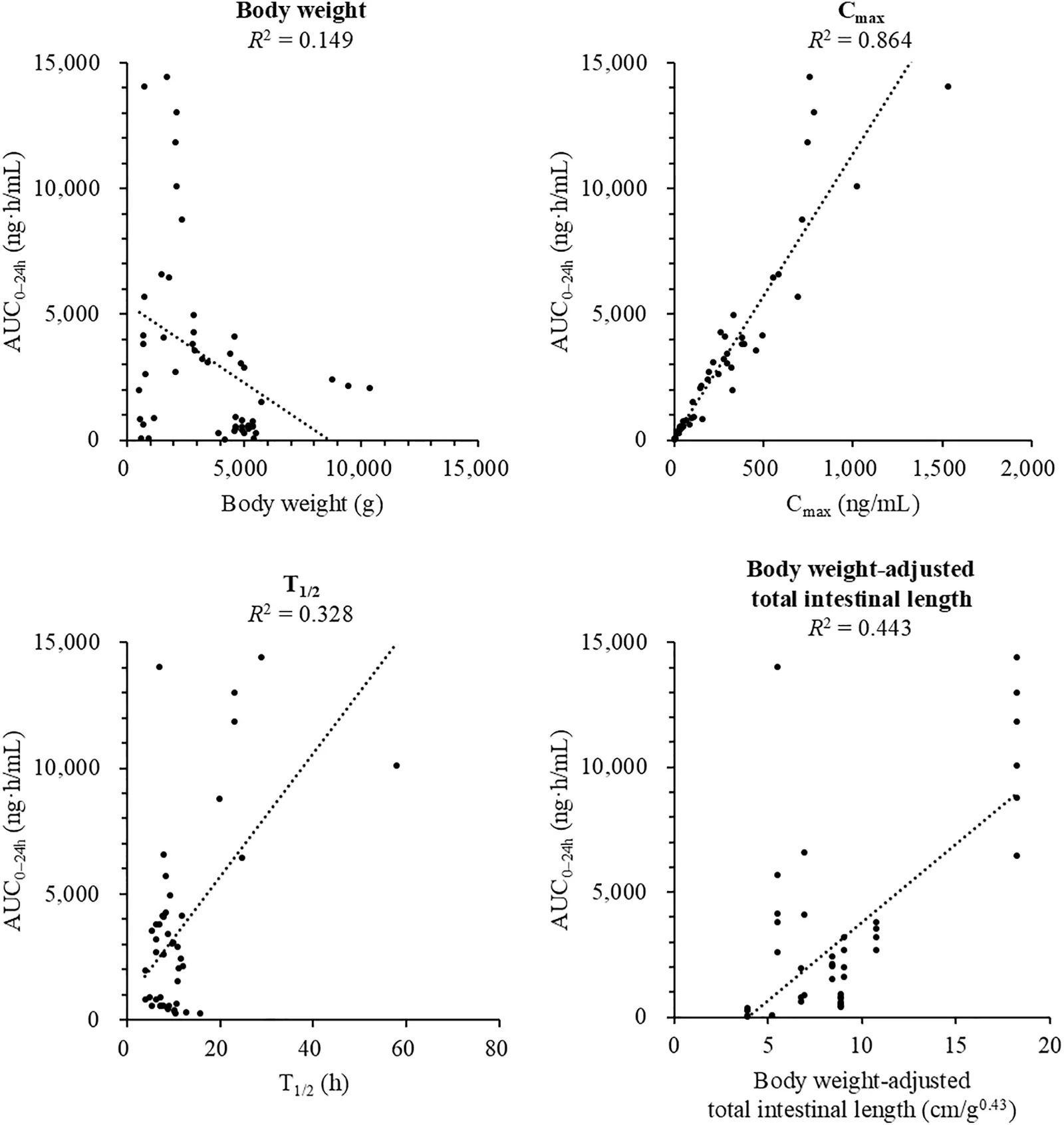
Correlation between area under the plasma concentration–time curve from 0 to 24 hours (AUC_0–24h_) of BXA, and body weight, maximum plasma concentration (C_max_) of BXA, elimination half-life (T_1/2_) of BXA, and body weight-adjusted total intestinal length following administration of baloxavir marboxil at 2.5 mg/kg. The dashed lines represent linear regression lines, and the coefficient of determination (*R*^2^) is indicated in each panel.

## Discussion

The use of anti-influenza drugs in captive rare birds may be considered both therapeutically, for infected individuals and metaphylactically, following detection of HPAIV infection within a flock or facility. Previous studies in HPAIV-infected ducks demonstrated that systemic BXA exposure is associated with reductions in mortality and virus shedding, providing a pharmacokinetic basis for defining effective antiviral exposure in birds. However, a lack of species-specific pharmacokinetic information has limited the practical use of these antivirals in various avian species. In the present study, the pharmacokinetics of BXM and BXA were systematically evaluated in rare avian species using administration methods applicable to zoological settings. The results demonstrated marked interspecies variability in systemic exposure to BXA. These findings provide a pharmacokinetic basis for species-optimized administration strategies and support the rational application of BXM in diverse avian species.

In the present study, the effects of different oral administration routes on the pharmacokinetics of BXA were evaluated in white-tailed eagles. Among the three administration methods examined, the highest BXA exposure was observed when BXM capsules were placed inside the rainbow trout (first administration). In contrast, no substantial differences in exposure were detected between forced administration of an oral capsule followed by trout feeding (second administration) and gastric tube administration of a BXM suspension (third administration). Although the BXA exposure after the second and third administrations was lower than that after the first administration, this was primarily driven by the exceptionally high AUC_0–24h_ accompanied by a high C_max_ observed in one individual during the first administration. In an additional analysis excluding this individual, the differences in AUC_0–24h_ were no longer statistically significant. Therefore, the higher BXA exposure observed after the first administration does not appear to represent a consistent effect of the administration method. The absence of a clear difference in exposure between the second and third administrations, which differed in whether BXM was mixed with food, suggests that the pronounced food-dependent reduction in BXM absorption reported in humans may be less pronounced in white-tailed eagles. Accordingly, when BXM is used to treat HPAIV infections in captive birds, ensuring adequate therapeutic exposure by any feasible method is more important than adhering to a standardized administration protocol.

Pharmacokinetic analyses demonstrated substantial interspecies variability in the systemic exposure to BXA across avian species. Even at the same administered dose, marked differences in BXA exposure were observed among the species, with substantial variations in pharmacokinetic parameters. Relatively low systemic exposure to BXA was observed in white-tailed eagles, puna ibises, peregrine falcons, Oriental storks, rock ptarmigans, and Amami jays. In contrast, exceptionally high exposure was observed in the Eurasian eagle-owl and southern rockhopper penguins. BXA exposure at doses of 2.5 and 12.5 mg/kg was compared among species to further characterize these differences. In Humboldt penguins, African penguins, puna ibises, and American flamingos, increasing the dose from 2.5 to 12.5 mg/kg resulted in less-than-proportional increases in C_max_ and AUC_0–24h_ of BXA, suggesting potential saturation of absorption. In contrast, C_max_ and AUC_0–24h_ of BXA increased in a more than dose-proportional manner when the dose was increased from 2.5 to 12.5 mg/kg, in common murres, great white pelicans, peregrine falcons, and Oriental storks, potentially due to saturation of first-pass metabolism and/or elimination pathways. In most of the species examined, the plasma concentrations of the prodrug BXM were minimal, suggesting its rapid conversion to BXA. However, relatively high plasma BXM concentrations were detected in Humboldt penguins and African penguins. Importantly, BXM was no longer detectable at 24 h after administration in any species or at any dose, indicating that the accumulation of the prodrug is likely limited. The elimination half-life of BXA also differed among species, with relatively short half-lives observed in puna ibises and American flamingos (5.5–6.1 h) and longer half-lives observed in southern rockhopper penguins (23.6–29.6 h). These values are intermediate between those reported in humans (48.9–114 h) and mice (2.2–3.1 h), suggesting that interspecies differences exist not only in absorption but also in metabolic and excretory processes [27,33].

Collectively, these findings highlighted species-specific variability across all pharmacokinetic phases, including absorption, metabolism, and elimination, and emphasized the need to establish suitable dosing regimens for individual avian species. Simultaneously, the observation of similar pharmacokinetic profiles in closely related species, such as Humboldt and African penguins, suggests that pharmacokinetic data from phylogenetically related species may be extrapolated when direct data are unavailable.

In a repeated-dose study conducted in a Eurasian eagle-owl, the C_max_ of BXA increased in a dose-dependent manner. The pharmacokinetic profiles following the first and seventh administrations were comparable, suggesting no obvious accumulation of BXA. Although these observations were limited to a single individual, they provide preliminary evidence that short-term repeated administration of BXM does not markedly alter the pharmacokinetics of BXA.

The factors influencing the AUC_0–24h_ of BXA were examined. C_max_ showed a strong correlation with AUC_0–24h_. Because C_max_ is a pharmacokinetic parameter that reflects drug absorption, this result suggests that species-specific differences strongly influence BXA exposure in birds due to differences in drug absorption characteristics. In contrast, body weight-adjusted total intestinal length showed only a weak positive correlation with the AUC_0–24h_. Total intestinal length per body weight is considered an indicator that may influence intestinal permeability and absorption efficiency in the intestinal tract; therefore, it may partly explain variation in BXA exposure among species. In mammals, BXM is a prodrug that is converted to BXA in the intestine and/or liver following oral administration and appears in plasma primarily as BXA [10]. Consequently, similar metabolic processes and subsequent exposure profiles are anticipated in avian species, indicating that interspecies differences in the expression levels or enzymatic activities of the esterases responsible could likewise influence BXA exposure and reduce the correlation with anatomical indices. The results of this study suggest that BXA exposure can be predicted, to a certain extent, when anatomical information is available. However, for more accurate predictions, detailed analyses of esterase expression levels and activities in the intestine and liver of each species are necessary.

In addition to absorption-related factors, elimination-related factors were examined. T_1/2_ showed only a weak association with the AUC_0–24h_. Previous studies reported that BXA is metabolized by UGT1A3 and CYP3A [27]. Therefore, to understand interspecies differences in T_1/2_ and improve the accuracy of pharmacokinetic predictions, it is important to clarify species differences in the expression and activity of these metabolic enzymes.

The pharmacokinetic target used to define the dosing regimens was based on the previous HPAIV-infected duck study. Although C_24h_ has been used as an important pharmacokinetic (PK)/pharmacodynamic (PD) index in mammalian models and human clinical development, PK/PD relationships established in mammalian influenza models may not directly translate to HPAIV infection in birds, in which systemic viral replication can occur. Therefore, the previous avian study directly compared several BXA pharmacokinetic parameters and identified AUC_0–24h_ as the parameter most strongly associated with a reduction in virus shedding. The BXA AUC_0–24h_ of 2,374 ng·h/mL achieved with the therapeutically effective 2.5 mg/kg regimen in ducks was consequently used as the reference exposure for defining the species-specific dosing regimens in the present study. Based on the obtained pharmacokinetic data, 2.5 mg/kg every 2 days was likely sufficient to achieve the BXA exposure level associated with antiviral efficacy in southern rockhopper penguins (Fig 5, S3 Table). In Humboldt penguins, African penguins, common murres, great white pelicans, American flamingos, black-faced spoonbills, and Eurasian eagle-owls, a once-daily dose of 2.5 mg/kg was expected to achieve the target exposure level. In contrast, puna ibises and Oriental storks may require 12.5 mg/kg once daily. Although a 12.5 mg/kg dose was not evaluated in white-tailed eagles, the higher BXA exposure observed at 2.5 mg/kg relative to that in Oriental storks suggests that a once-daily dose of 12.5 mg/kg would represent a reasonable regimen. For rock ptarmigans, administration of 12.5 mg/kg twice daily appears to be necessary to achieve the target exposure. In peregrine falcons and Amami jays, however, even 12.5 mg/kg administered twice daily was considered unlikely to achieve sufficient exposure to confer antiviral efficacy. Similarly, in Okinawa rails, which previously showed low exposure following oral administration, subcutaneous administration has been explored as an alternative delivery route [32]. These findings suggest that alternative administration routes, such as subcutaneous administration, may warrant consideration in avian species with poor BXA exposure following oral BXM administration.

**Fig 5:**
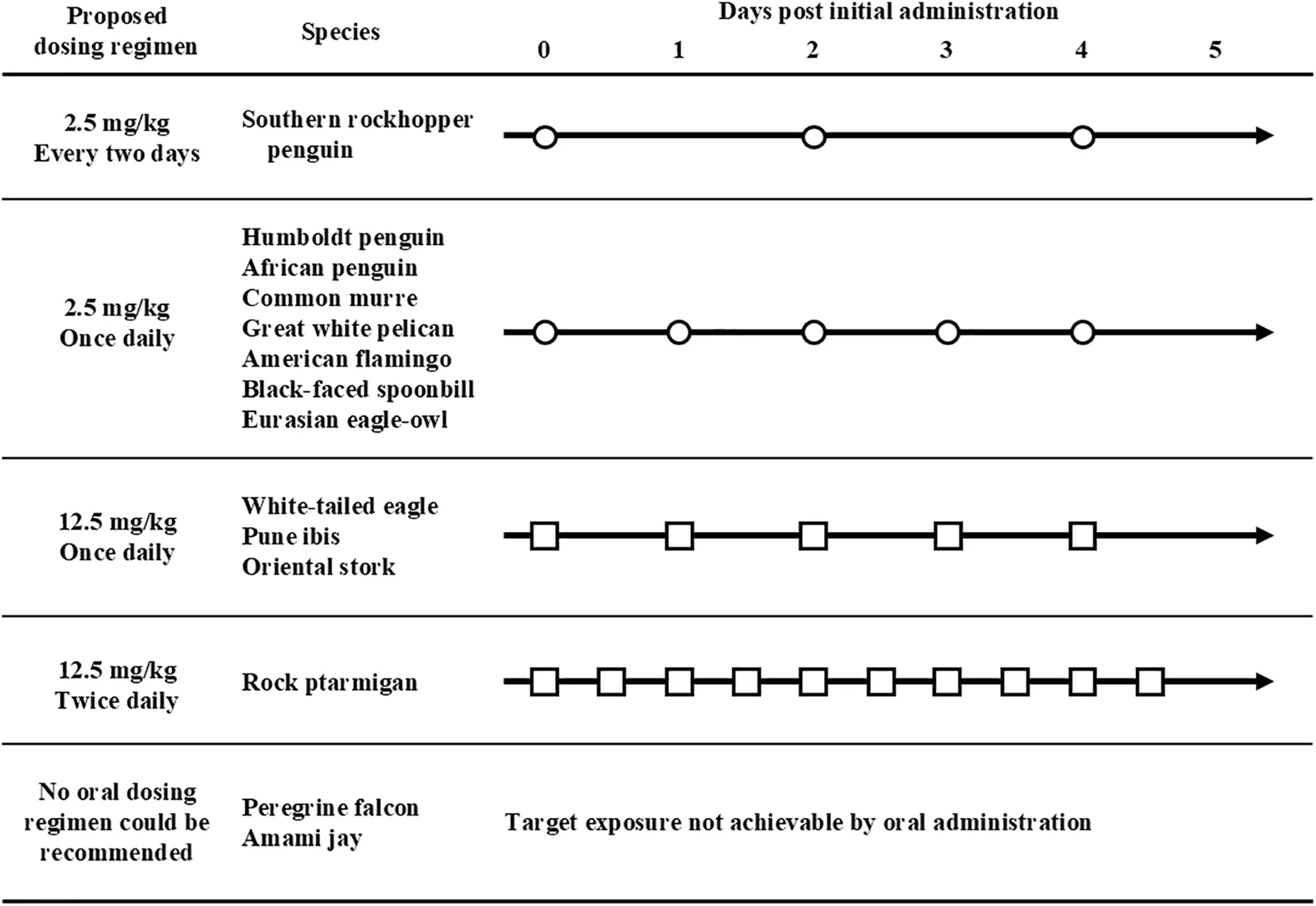
Proposed dosing regimens of baloxavir marboxil in birds to maintain effective baloxavir acid exposure against high pathogenicity avian influenza virus. Each row represents the proposed dose and dosing interval for the indicated species over a representative treatment course.

A concern for the veterinary use of BXM in rare avian species is the potential emergence of antiviral resistance. In an H5N1-infected mouse model, delayed initiation of BXM treatment resulted in the selection of viruses with reduced susceptibility to baloxavir, including variants carrying the PA-I38T substitution [34]. Similarly, in a previous study using HPAIV-infected ducks, virus shedding initially decreased after the initiation of BXM treatment but subsequently rebounded in some birds, with no viral sequence analysis performed in this study [21]. Despite this rebound, virus shedding eventually ceased during continued administration over the scheduled five-day treatment course. The five-day treatment course proposed in the present study follows the regimen shown to be therapeutically effective in the previous duck study, in which repeated administration was used to maintain adequate BXA exposure during the acute phase of infection. After completion of the treatment course, treated birds should continue to be maintained under strict isolation and biosecurity conditions, with continued clinical monitoring, until the risk of onward transmission is considered sufficiently low.

## Conclusion

This study provides the first systematic pharmacokinetic characterization of BXM and BXA in multiple avian species under zoological conditions. These results highlight the substantial interspecies variability in systemic exposure to BXA and demonstrate that species-specific optimization of dosing strategies is required to achieve exposure levels associated with antiviral efficacy. These findings establish a pharmacokinetic framework for the rational use of BXM in the management of HPAIV infection in captive rare birds and provide a basis for designing practical treatment protocols across diverse avian species. Nevertheless, to mitigate the risk of antiviral resistance, BXM must be strictly restricted to captive rare birds and should not be used broadly in avian populations.

## Supporting information

S1 Fig Plasma concentration–time profiles and pharmacokinetic parameters of baloxavir acid (BXA) in a Eurasian eagle-owl repeatedly administered baloxavir marboxil at doses of 2.5, 5.0, and 10 mg/kg. Concentrations following the first and seventh administrations are shown. ND, not determined.Plasma concentration–time profiles and pharmacokinetic parameters of baloxavir acid (BXA) in a Eurasian eagle-owl repeatedly administered baloxavir marboxil at doses of 2.5, 5.0, and 10 mg/kg.
(DOCX)

S1 Table Individual information for birds administered baloxavir marboxil. *: Estimated age, ND: Not determined, M: Male, F: Female.
(XLSX)

S2 Table Results of hematological and hematochemical analysis of birds administered baloxavir marboxil. Hct, hematocrit; AST, aspartate aminotransferase (formerly glutamic oxaloacetic transaminase, GOT); ALT, alanine aminotransferase (formerly glutamic pyruvic transaminase, GPT); UA, uric acid; BA, bile acids; Alb, albumin; TP, total protein; IP, inorganic phosphorus; Na, sodium; K, potassium; Ca, calcium; Glu, glucose; ND, Not determined.
(XLSX)

S3 Table Proposed dosing regimens of baloxavir marboxil based on target exposure of baloxavir acid. AUC_0–24h_, AUC_0–12h_, and AUC_24–48h_, area under the plasma concentration–time curve of BXA from 0–24, 0–12, and 24–48 hours after BXM administration, respectively.
(XLSX)
