# Supplementary material for: Pharmacokinetic diversity of baloxavir marboxil across rare avian species
Source: PLoS One. 2026 Oct 23;21(10):e0361119. doi: 10.1371/journal.pone.0361119 (PMC13693923; doi:10.1371/journal.pone.0361119)
Supplement: Supplementary file 1 [file pone.0361119.s001.docx]

**Fig. S1. Plasma concentration–time profiles and pharmacokinetic parameters of baloxavir acid (BXA) in a Eurasian eagle-owl repeatedly administered baloxavir marboxil at doses of 2.5, 5.0, and 10 mg/kg.** Concentrations following the first and seventh administrations are shown. ND, not determined.
